# Metal exposure of workers during recycling of electronic waste: a cross-sectional study in sheltered workshops in Germany

**DOI:** 10.1007/s00420-021-01651-9

**Published:** 2021-01-24

**Authors:** Johannes Gerding, Claudia Peters, Wolfgang Wegscheider, Johanna Stranzinger, Frederik Lessmann, Katrin Pitzke, Volker Harth, Udo Eickmann, Albert Nienhaus

**Affiliations:** 1 German Social Accident Insurance, Institution for the Health and Welfare Services (BGW), Department for Occupational Medicine, Hazardous Substances and Public Health, Hamburg, Germany; 2 University Medical Centre Hamburg-Eppendorf (UKE), Competence Centre for Epidemiology and Health Services Research for Healthcare Professionals (CVcare), Hamburg, Germany; 3 University Medical Centre Hamburg-Eppendorf (UKE), Institute for Occupational and Maritime Medicine (ZfAM), Hamburg, Germany; 4 Institute for Occupational Health and Safety (IFA) of the German Social Accident Insurance (DGUV), Sankt Augustin, Germany

**Keywords:** Biomonitoring, Air monitoring, Metal exposure, Electronic waste, Sheltered workshop, Urine

## Abstract

**Objectives:**

In Germany, the initial step of electronic waste (e-waste) recycling frequently takes place in sheltered workshops for physically and mentally handicapped workers (*Werkstätten für behinderte Menschen (WfbM)*, in german language). E-waste recycling involves a potential risk of exposure to toxic metals. Therefore, we assessed the occupational exposure of recycling workers to toxic metals to identify potential health risks and insufficient protective measures.

**Methods:**

We used a combined air- and bio-monitoring approach to determine exposure of recycling workers to toxic metals. Air and urine samples were collected in five sheltered workshops in Germany and were analysed for their content of aluminium, antimony, arsenic, beryllium, cadmium, chromium, cobalt, mercury and nickel. Results were compared to German and international occupational limit values and to metal exposures of workers in conventional e-waste recycling firms.

**Results:**

Exposure of recycling workers in five German sheltered workshops to the studied metals and their compounds was below German and international occupational limit values across all facilities studied considering both air and urine samples. Workers in the present study were not exposed to higher amounts of toxic metals than workers in conventional e-waste recycling firms.

**Conclusion:**

This is the first study on toxic metal exposure of recycling workers in sheltered workshops. The results of this study revealed a low occupational exposure of e-waste recycling workers to toxic metals in this type of enterprises. Current work methods and safety measures provide the workers with adequate protection.

**Supplementary Information:**

The online version contains supplementary material available at 10.1007/s00420-021-01651-9.

## Introduction

Around the world, 20–50 million tons of electronic waste (e-waste) are generated each year. Electronic components contain a variety of harmful metals to which employees may be exposed during recycling. Examples of these include aluminium (Al), antimony (Sb), arsenic (As), beryllium (Be), cadmium (Cd), chromium (Cr), cobalt (Co), mercury (Hg) and nickel (Ni). According to studies, just 10% of the electronic waste generated worldwide is recycled under conditions where the health of workers is effectively protected (Julander et al. 2014).

Workers may also be exposed to metals in the recycling of tube and flat-screen displays. For example, first-generation LCD flat-screen displays use Hg-based gas discharge lamps (CCFL) for backlighting. CFFLs may fracture during manual dismantling of such devices (Julander et al. 2014). Other metals are present in cathode-ray tubes or circuit boards (Thullner et al. 2013). In Germany, some disassembly of displays is performed in sheltered workshops (*Werkstätten für behinderte Menschen (WfbM)*, in german language). These facilities have been established to reintegrate people with physical, mental or psychiatric disorders into working life. In sheltered workshops, the workplace and work activities are adapted to the special needs of the workers. Knowledge of the occupational exposure of workers in such facilities to toxic metals is limited. The aim of this study was to assess the metal exposure of recycling workers involved in the disassembly of e-waste to identify potential health risks and to assess the sufficiency of existing protective measures.

## Materials and methods

### Study design and study population

In a cross-sectional study, toxic metal exposure occurring during the disassembly of e-waste was studied using a combined air- and biomonitoring approach (analysis of urine samples). Results were subjected to a statistical analysis and related to german and international occupational exposure limit values. Activities covered by the present study included disassembly of flat-screen and tube displays (CRT & LCD displays) as well as other disassembly and sorting activities related to recycling of small electronic devices (consumer electronics). Exposure of 71 workers to toxic metals was studied between July 2017 and January 2018 at workplaces of five sheltered workshops in Germany. In addition to the 51 workers directly involved in e-waste recycling (exposed workers), 20 office workers of the facilities were included in the study as a non-exposed control group. All participants completed a brief questionnaire with demographic information and questions on work tasks and potential background exposures (e.g. from dental amalgam fillings, tobacco and fish consumption). Participation in the study was voluntary. The participants (and their legal representative, if applicable) were informed about the background and objectives of the study and gave their written consent to participate in the study. Biomonitoring samples and questionnaires were number encoded to ensure the anonymity of participants. The study was conducted in accordance with the requirements of the data protection legislation. The study design was reviewed and approved by the Hamburg Ethics Commission.

### Collection of samples

#### Sample collection

In the e-waste recycling areas of the workshops, air samples were collected under standard operating conditions during disassembly work to determine the air concentrations of metal dusts and Hg. Work methods and material throughput rates were documented. Air samples were taken on two consecutive days. Measurement points, measurement methods and work processes remained unchanged during sampling.

Dust samples were collected in the breathing zone of the workers (personal sampling) and by stationary sampling next to the workplaces as described below. Both the inhalable dust fraction (*n* = 4, only pilot measurements) and the respirable dust fraction (*n* = 40) were sampled by personal sampling. Stationary samples (inhalable (*n* = 21) and respirable (*n* = 12) dust fraction) were taken at comparable positions across all facilities. Two environmental control samples were collected in the office areas of the facilities.

Hg was sampled similarly to dust samples by personal (*n* = 31) and stationary (*n* = 21) sampling according to methods described below.

Spot urine samples of recycling workers (*n* = 51) and office workers without e-waste recycling activities (*n* = 20) were collected at the end of shifts.

#### Air samples

Air samples were collected using a validated method according to air monitoring methods 7808 (metal dust) and 8530 (Hg, workshops A–C) of the Institute for Occupational Safety and Health of the German Social Accident Insurance (IFA) and according to a validated method of Hebisch (Hebisch et al. 2018). Inhalable and respirable dust fractions were collected by personal and stationary sampling at the workshops and in office areas.

The inhalable and respirable metal dust fractions were collected using a personal air sampling (PAS) pump (flow rate 10 l/min) and an inhalable dust sampling system (inhalable metal dust fraction—GSP) or a respirable dust sampling system (respirable metal dust fraction—FSP) on a membrane filter in a filter cassette (37 mm diameter, pore width 8 µm) and were analysed in the laboratory.

Hg was sampled using a personal air sampling (PAS) pump with hopcalite/quartz fibre tubes (flow rate 1.0 l/min: facilities A–C) or activated charcoal tubes (Anasorb C300, 200 mg activated charcoal, SKC Inc. eighty four, pa, USA, flow rate 0.25 l/min: facilities D, E) and were analysed in the laboratory.

Sample collection was carried out task specific during a sampling duration of 4 to 6 h per sample.

#### Biomonitoring

Spot urine samples were collected in 250 mL polypropylene containers, pre-rinsed with 1 M nitric acid to avoid external contamination, on the day of the air sample collection after the end of the shift in the middle of the working week. Prior to sample collection, study participants were asked to change clothes and wash hands. The collection of the urine samples was organised by the facility’s medical service. The samples were stored and shipped at − 20 °C until analysis.

### Laboratory analytics

#### Air samples

Sample analysis included the metals Al, Sb, Be, As, Cd, Cr, Co and Ni and was performed at the laboratories of the IFA in accordance with the validated IFA method 7808 “Metals and their compounds (ICP mass spectrometry)”. Samples were prepared for analysis using an open-vessel acid digestion (2 parts by volume 65% nitric acid, 1 part by volume 25% hydrochloric acid) and were quantified by inductively coupled plasma mass spectrometry (ICP-MS). Please refer to the supplementary material for details on the method and the validation parameters.

The Hg analysis of samples from facilities A–C was carried out in accordance with IFA method 8530 using atomic fluorescence spectrometry. Due to lasting material supply limitations, samples from facilities D and E were analysed for Hg using atomic absorption spectrometry in accordance with the method of Hebisch (Hebisch et al. 2018).

The limit of quantification (LOQ) of Al, Sb, Be, As, Cd, Cr, Co and Ni was determined according to the blank value method of DIN EN 32645. The LOQ was calculated as ten times the standard deviation of blank samples. The limit of detection (LOD) was calculated as three times the standard deviation of blank samples.

LOQ and LOD of Hg were determined by the calibration method of DIN EN 32,645 (LOQ: coverage factor k = 3; n-2 degrees of freedom; P = 0,995, two-sided. LOD: t(f) = n-2; P = 0,99, one-sided).

The limits of quantification ranged from 0.0017 to 0.39 µg/m^3^ depending on the element and the air volume sampled (see Table 1).

**Table 1:** Limits of detection (LOD) and quantification (LOQ) of the analytical air monitoring (µg/m^3^) and biomonitoring (µg/l) methods

	Air monitoring	Biomonitoring
	LOQ^*^ [µg/m^3^]	LOQ (LOD) [µg/l]
Aluminium	0.17	3.0 (1.0)
Antimony	0.17	1.0 (0.3)
Arsenic	0.027	6.0 (2.0)
Beryllium	0.0017	0.09 (0.03)
Cadmium	0.017	0.50 (0.16)
Chromium	0.21	0.50 (0.16)
Cobalt	0.083	1.0 (0.3)
Nickel	0.39	1.0 (0.3)
Mercury	0.013; 0.02^#^	0.6 (0.2)

Detailed description of methods for LOD/LOQ determination in the “Laboratory analytics” section

*Air monitoring LOQs depend on the air volume sampled (standard 10 l/min, 2 h) and may differ on a daily basis e.g. in case of prolonged sampling times (4 to 6 h in the present study)

^#^Different LOQ for mercury: facilities D and E

#### Biomonitoring

Chemicals: single element stock solutions (c = 1 mg/L) were purchased from Baker. Triton-X100 was purchased from Fluka. Nitric acid, acetic acid, and palladium modifier were purchased from Merck. All chemicals and reagents used were at least p.a. grade. Ultra-pure water (R > 18 MΩ) was obtained from a Merck Milli-Q Reference Plus system.

All biomonitoring analyses were performed at the Institute for Occupational and Maritime Medicine, Hamburg. Sb, As, Cd, Hg, Cr, Co, Ni, Al and Be were determined by in-house methods that are generally based on the methods published by the German MAK Commission working group “Analyses of hazardous substances in biological materials” (DFG 2002). Sb, As and Hg were determined by cold-vapour atomic absorption spectroscopy (CV-AAS) on a Thermo Fisher ICE3500 and calibration was performed with a solvent-based external calibration. The other elements (Cd, Cr, Co, Ni, Al and Be) were determined by graphite-furnace atomic absorption spectroscopy (GF-AAS) on a Varian SpectrAA 800 and calibration was performed with a matrix-based external calibration.

The limits of quantification (determined by ten-times the standard deviation of ten solvent blank samples) ranged from 0.09 to 3.0 µg/L depending on the element. The limits of detection (determined by three-times the standard deviation of ten solvent blank samples) ranged from 0.03 to 1.0 µg/L (limits of detection and quantification in Table 1).

Urinary creatinine was determined by reversed phase high performance liquid chromatography with ultraviolet detection (HPLC–UV) on a VWR Elite LaChrom system. Calibration was conducted by external calibration ranging from 0.05 to 3.0 g/L. The limit of quantification (based on a signal to noise ratio of nine) was 0.03 g/L).

For internal quality control, quality control samples in high and low levels (Q high and Q low) prepared in-house were analysed within each batch of study samples. Deviations from target values < 15% were regarded tolerable. External quality control was conducted by regular successful participation in the German external quality assurance scheme (G-EQUAS) organised by the German Society for Occupational Medicine and Environmental Medicine (DGAUM e.V.).

### Statistical analysis

The statistical analysis for the description of the study population (Table 2) was performed using descriptive statistics. The results were stated as absolute and relative numerical values. The group comparison was performed using the Chi-square test based on Pearson, or where cell frequency was low, using Fisher’s exact test. For the air monitoring and biomonitoring analyses, descriptive statistical methods were used and key parameters such as the number of measurements (N), range, median and geometric mean (GM) were shown for the substance concentrations. To test differences between the recycling workers (exposed workers) and office workers (control group) for the biomonitoring results, the Mann–Whitney *U* test was used for independent samples. The significance level was set at *p* < 0.05. The analyses were performed using IBM SPSS Statistics 26.

**Table 2:** Statistical data of recycling workers and controls from five e-waste recycling sheltered workshops in Germany

Characteristics	Recycling workers (*n* = 51)	Controls (*n* = 20)	*p* value
Age in years			
Range	24–76	27–59	0.22
Mean/median	46.2 / 49.0	50.6 / 54.5	
	*n* (%)	*n* (%)	
Sex			
Female	4 (7.8)	5 (25.0)	0.13
Male	46 (90.2)	15 (75.0)	
No answers	1 (2.0)	0	
*Work tasks*			
Flat screen dismantling	14 (27.5)	0	NA
Tube screen dismantling	24 (47.1)	0	NA
E-waste recycling	48 (94.1)	0	NA
*Non-occupational factors*			
Dental amalgam fillings	23 (45.1)	7 (35.0)	0.59
Smoking			
Smoker	29 (65.9)	4 (20.0)	0.008
Non-smoker	22 (43.1)	16 (80.0)	
Fish consumption (last 48 h)	4 (7.8)	3 (15.0)	0.55
Frequency of fish consumption			
1–2 times a week	26 (51.0)	7 (35.0)	0.04
1–3 times a month	18 (35.3)	13 (65.0)	
Never	7 (13.9)	0	

*NA* not available

### Comparison of exposure data with occupational exposure levels

The results of the air measurements were placed in the context of health based occupational limit values (OEL) as specified in the German/European occupational health & safety regulation as time-weighted average (TWA) 8 h/shift). These are OEL based on the German technical rule for hazardous substances (TRGS) 900 (AGW), Committee on Hazardous Substances 2006). Exposure to carcinogenic metals was compared to Risk based OELs (acceptable concentrations) according to TRGS 910 (Committee on Hazardous Substances 2014). In the absence of a german OEL, an international limit value was used for Sb (Austrian Federal Ministry of Labour 2018). The results of biomonitoring measurements (95.th percentile) were placed in the context of German occupational health & safety standards. These are health based biological limit values (BGW) set by TRGS 903 (Committee on Hazardous Substances 2013). In the absence of a health based BGW, we used the biological working substance reference values (BAR) and biological guideline values (BLW) of the German Commission for the Study of Working Materials Harmful to Health (MAK Commission 2019) as exposure benchmarks. BAR and BLW summarize statistical data on urinary metal excretion of workers at other workplaces (BLW) or the 95th percentile of the general population (BAR). They are no health based exposure limits.

## Results

A total of 71 workers in five sheltered workshops (facilities A–E) participated in our study. 51 workers (exposed personnel) worked in the recycling area (8 to 13 recycling workers per facility). Another 20 participants worked in the administrative/office areas (control group) (Table 2). The median age of the exposed group was five years less than that of the control group. Electrical equipment disassembly (not including the disassembly of displays) was quoted as the most common work activity of 94% of the survey respondents, while just under 50% of the participants performed the disassembly of tube displays and 28% worked in the disassembly of flat-screen displays. Multiple answers were possible and workers usually did not specify only one work activity. The share of smokers (*p* = 0.008 Fisher’s exact test) and frequency of fish consumption (*p* = 0.04 Chi-square test) was higher in the group of recycling workers compared to the control group. There was no difference in the presence of amalgam dental fillings.

### Documentation of exposure parameters

The recycling halls of the facilities were located on the ground floor with doors to adjacent rooms and automated gates to the outside. The workshops were not equipped with technical ventilation systems or local exhaust ventilation devices. Ventilation was provided only by tilting the windows and by opening the automated gates several times a day for transport activities. All facilities had a disassembly line for the disassembly of CRT devices, LCD devices and other electrical and electronic devices. The devices were stored outside on covered ground before and after disassembly. The disassembly work was performed in the facilities simultaneously at between 5 and 20 workstations. The workers did not wear personal protective equipment apart from work gloves during handling of sharp objects.

The CRT devices were dusted off in four of the five facilities using compressed air in a closed dust removal booth (equipped with a ventilation system in facilities B, C and D) or cleaned using an industrial vacuum cleaner (facility E). No prior cleaning was done in facility A. At the disassembly site, the display tubes of the devices were first vented with a rod (facility D: endpiece removed with a hammer), then disassembled using manual tools. Disassembly of a CRT device took around 5–25 min, including material and equipment transport. The LCD devices were placed on the work surface and disassembled using power tools. When the LCD panel was opened, the CCFL tubes, which contain Hg, were removed. The dismantled tubes were placed in open containers or at the workstation and brought to collection bins for (destructed) tubes after the device had been disassembled. In facility A, the dismantled tubes remained in an open plastic container next to the work surface until the end of the shift. Disassembly of a LCD device took around 11–35 min on average including set-up times. In addition to the disassembly of CRT and LCD devices, other consumer electronic goods were dismantled at other workstations in the workshops.

### Biomonitoring

Urine samples from 71 employees were available for analysis (recycling workers *n* = 51, control group *n* = 20). Three samples from the recycling workers group were not analysed for Sb, As and Cd due to the low available sample volume.

**Fig. 1:**
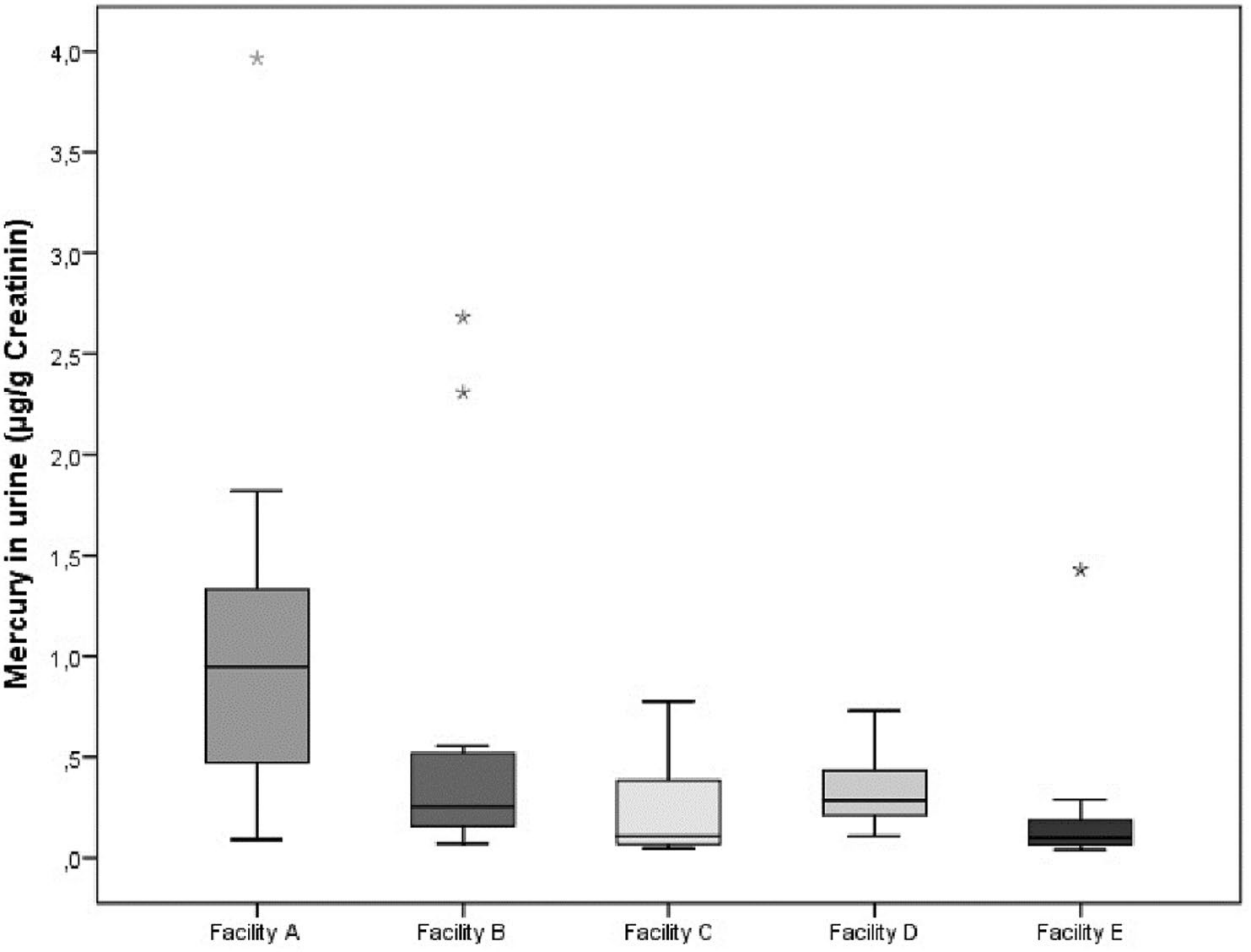
Distribution of mercury concentrations (µg/g creatinine) in urine samples of recycling workers from five german e-waste recycling sheltered workshops (facilities A–E). The boxplot is divided by the median. The box contains the middle 50% of the distribution (interquartile range) and the whiskers show 25% each. Outliers are shown separately. No further statistical test were conducted due to the limited number of samples

Table 3 summarizes the urinary metal concentrations in samples of recycling workers and the control group. The metals Al and Ni were found in over 90% of the samples above the LOD in both groups. As, Hg and Co were present in over 50% of the urine samples. The metals Sb, Cd and Cr were detected less frequently (< 50%). Be was only detected in five samples (7%). A statistically significant difference was found between the Hg concentrations in samples of e-waste recycling workers compared to the control group. The breakdown of results by facility reveals higher Hg concentrations in urine samples of recycling workers in facility A (Fig. 1). The workers in facility E were also exposed to higher concentrations of Sb (GM 1.04 µg/l) than the workers in the other facilities (GM 0.22 µg/l). Urine samples of the smokers exhibited higher concentrations of Cd (GM 0.25 µg/l, *n* = 29) than samples of non-smokers (GM 0.09 µg/l, *n* = 22). There were no other differences in the excretion profile of the biomarkers, neither between the recycling workers and officer workers, nor among the facilities.

**Table 3:** Metal concentrations in urine samples (µg/l and µg/g creatinine (as µg/g)) of recycling workers and the control group of five e-waste recycling sheltered workshops in Germany

	Recycling workers					Controls					
Metal*	*n*	Range	Median	GM	95th percentile	*n*	Range	Median	GM	95th percentile	*p* value
Aluminium (µg/g)	51	0.85–39.3	3.40	3.18	9.8	20	1.0–15.7	2.90	2.89	15.3	0.6
Aluminium (µg/l)	51	1.5–21.2	4.10	3.79	12.3	20	1.5–8.7	3.60	3.28	8.7	0.4
Antimony (µg/l)	49	0.15–2.4	0.15	0.26	2.0	19	0.15–2.2	0.50	0.36	n.a	0.2
Arsenic (µg/l)	50	1.0–8.6	3.0	1.96	7.0	20	1.0–10.0	3.00	2.05	9.7	0.8
Beryllium (µg/l)	51	0.02–0.05	0.02	0.02	0.05	20	0.02–0.05	0.02	0.02	0.05	n.a
Cadmium (µg/l)	51	0.08–1.8	0.08	0.16	1.3	20	0.08–1.3	0.08	0.13	1.3	0.5
Chromium (µg/g)	51	0.02–0.64	0.08	0.09	0.5	20	0.02–0.7	0.07	0.09	0.6	0.9
Chromium (µg/l)	51	0.08–1.1	0.08	0.10	0.25	20	0.08–0.25	0.08	0.10	0.25	0.9
Cobalt (µg/l)	51	0.15–1.6	0.5	0.32	1.1	20	0.15–0.5	0.50	0.29	0.5	0.7
Nickel (µg/l)	51	0.15–3.8	0.5	0.74	2.9	20	0.15–3.0	0.50	0.53	2.9	0.1
Mercury (µg/g)	51	0.05–4.0	0.30	0.32	2.0	20	0.04–2.7	0.20	0.18	2.6	0.06
Mercury (µg/l)	51	0.1–4.6	0.30	0.38	2.7	20	0.1–3.7	0.10	0.21	3.6	0.03

Creatinine-adjusted concentrations are only shown for Al, Cr and Hg if required for comparison with creatinine-adjusted biologic limit values (see Table 6)

*When calculating the statistical location parameters median and geometric mean (GM), samples below the limit of detection (LOD) were assigned a numerical value of 50% of the LOD. Similarly, samples with analyte concentrations below the limit of quantification (LOQ) were allocated a value of 50% of the LOQ. Details on LOD and LOQ in Table 1

### Air samples

The concentration of nine metals was determined in the air of five e-waste processing sheltered workshops. Al, Sb, As, Be, Cd, Cr, Co and Ni concentrations were only determined in the inhalable dust fractions from the breathing zone of the recycling workers, since pilot measurements revealed low metal concentrations in the in the respirable air samples collected (*n* = 4, see Table 4). A stationary sampling in the close proximity of recycling workplaces additionally covered the metal concentrations in both the inhalable and respirable dust fractions. Air concentrations of Hg were determined concurrently (personal and stationary samples). Results are summarized in Tables 4 and 5. Two environmental control samples from office areas of each building indicated no elevated background exposure (data not shown). The metals Al, Cr, Ni and Hg were frequently found in the personal and stationary samples (Al: 100%, Cr: 68%, Ni: 95%, Hg 95%). Sb (43%), As (8%), Cd (30%) and Co (20%) were detected less frequently. Be was not detected in any air sample. Workers in facility A were exposed to higher amounts of Hg (GM: 1.14 µg/m^3^) than the workers in the other facilities (GM: 0.33 µg/m^3^). The recycling workers in facility E were also exposed to higher Sb concentrations in the air (GM: 0.11 µg/m^3^) than workers in facilities A–D (GM: 0.07 µg/m^3^). Comparison of groups revealed no further differences in metal exposure of workers between the facilities. Metal concentrations were higher in samples from personal sampling compared to stationary sampling (up to a factor of two, see Table 5).

**Table 4:** Metal concentrations (µg/m^3^) in the respirable fraction samples of recycling workers in five german e-waste recycling sheltered workshops collected with personal and stationary sampling systems

	Recycling workers Personal Sampling		Recycling workers Stationary Sampling	
Metal [µg/m^3^]*	n	Median (GM) Range	n	Median (GM) Range
Aluminium	4	1.8 (1.74) 1.4–2.1	12	1.50 (1.62) 0.75–4.5
Antimony	4	0.077 (0.076) 0.064–0.088	12	0.062 (0.064) 0.047–0.14
Arsenic	4	0.026 (0.025) 0.022–0.029	12	0.032 (0.033) 0.021–0.077
Beryllium	NA		NA	
Cadmium	4	0.0082 (0.0081) 0.0068–0.0094	12	0.0068 (0.0074) 0.005–0.015
Chromium	4	0.079 (0.077) 0.065–0.09	12	0.12 (0.11) 0.065–0.24
Cobalt	4	0.022 (0.021) 0.018–0.024	12	0.034 (0.034) 0.018–0.062
Nickel	4	0.077 (0.076) 0.064–0.088	12	0.084 (0.08) 0.047–0.20
Mercury	NA		NA	

Personal sampling of the respirable dust fraction was only conducted as part of a pilot study (see “Results”)

*NA* not available

During calculation of the statistical location parameters median and geometric mean (GM), samples beneath the limit of quantification (LOQ) were assigned a numerical value equal to the LOQ. Refer to Table 1 for the LOQs

**Table 5:** Metal concentrations (µg/m^3^) in the inhalable fraction samples of recycling workers in five German e-waste recycling sheltered workshops collected with personal and stationary sampling systems

Metal [µg/m^3^]	Recycling workers Personal sampling		Recycling workers Stationary sampling	
	*n*	Median (GM) Range	*n*	Median (GM) Range
Aluminium	40	6.85 (8.23) 2.0–85.0	21	3.70 (3.70) 0.89–15.0
Antimony	40	0.075 (0.091) 0.051–0.34	21	0.062 (0.067) 0.047–0.17
Arsenic	40	0.033 (0.033) 0.021–0.069	21	0.031 (0.031) 0.018–0.13
Beryllium	NA		14	0.0015 (0.0015) 0.0013–0.0017
Cadmium	40	0.013 (0.017) 0.005–0.23	21	0.008 (0.009) 0.006–0.044
Chromium	40	0.16 (0.20) 0.081–1.4	21	0.12 (0.12) 0.063–0.64
Cobalt	40	0.035 (0.041) 0.018–0.31	21	0.033 (0.035) 0.015–0.18
Nickel	40	0.23 (0.27) 0.058–1.9	21	0.099 (0.11) 0.052–0.69
Mercury	31	0.44 (0.47) 0.14–3.3	22	0.31 (0.46) 0.16–6.6

During calculation of the statistical location parameters median and geometric mean (GM), samples below the limit of quantification (LOQ) were assigned a numerical value equal to the LOQ. Refer to Table 1 for the LOQs

*NA* not available

## Discussion

This is the first study to systematically examine occupational exposure to toxic metals among workers during the disassembly of consumer electronics in sheltered workshops. Toxic metals were only present in low concentrations in the air of workshops and in urine samples of workers.

With the exception of Be, all metals were detected with varying frequencies and levels of exposure, both in the air and urine samples. While the metals Al, Ni and Hg were frequently detected, Sb, As, Cd, Cr and Co were present in around 50% or less of the urine samples. Similarly, Al, Cr, Ni and Hg were present in over 70% of the air samples, while the other metals were detected in less than 50% of the samples. Be was not detected in any air sample above the limit of detection. The results of the air measurements and the human biomonitoring revealed variations in metal exposures among workers of the different facilities. The recycling workers in facility E were exposed to higher air concentrations of Sb than workers in facilities A–D (GM: 0.11 µg/m^3^ in facility E compared to 0.07 µg/m^3^ in facilities A–D). Workers in facility A were exposed to higher air concentrations of Hg (GM: 1.14 µg/m^3^ in facility A compared to 0.33 µg/m^3^ in facilities B–E). In both cases, elevated levels of Sb and Hg in urine samples of the workers were reported (Sb: 1.04 µg/l in facility E compared to 0.22 µg/l in facilities A–D. Hg: 1.02 µg/g creatinine in facility A compared to 0.22 µg/g creatinine in facilities B–E (geometric means)). The use of different occupation safety measures may explain these elevated Sb and Hg levels. For instance, the pre-cleaning of CFT displays was not carried out in a closed booth equipped with dust extraction in facility E, but rather using a simple industrial vacuum cleaner. This enables dusts containing Sb to be dispersed into the ambient air. In facility A, the dismantled CCFL tubes were placed in an open plastic box next to the work surface during the disassembly of LCD devices until the end of the shift. Elevated concentrations of Hg in the ambient air (and consequently the urine samples) may result from this modified workflow. Beyond this, there are no significant differences in terms of metal exposure in workers among facilities and among the different worker groups involved in recycling and office activities.

Compared to German and international occupational exposure limits (OELs), the metal exposure of recycling workers in this study was low. None of the German or international OELs were exceeded at the studied workplaces (see Table 6). These findings were confirmed by the results of the urine sample analysis. Binding occupational exposure limits (biological limit values) are only available for Al and Hg. None of the urine samples in this study exceeded the binding German biological limit values (BGW) of the metals Al and Hg (see Table 6). The geometric mean values of the urine concentrations of the other metals were also consistently below or within the range of non-binding biological working substance reference values. Some biomonitoring results for Sb and Cd exceeded the biological working substance reference values (BAR) significantly (factor 12 Sb, factor 2 Cd, see Table 6). In the general population, urinary Sb excretion is low (0.2 µg/L, Heitland (2006), Hoet (2013), 95.th percentile). Some urine samples of recycling workers showed Sb-concentration of up to 2.03 µg/L (2.4 µg/g creatinine). These values are elevated compared to the general population, but within the range of earlier studies on non-occupationally exposed persons (up to 3.4 µg/g creatinine in unexposed children according to Dezateux (1997). Our findings of elevated urinary Cd concentrations (above the limit value (BAR) of 0.8 µg/L) can be attributed to smokers. Smokers usually have a higher urinary Cd-excretion than nonsmokers. Becker (2003) reported on urinary Cd concentrations in smokers of 1.39 µg/L (95.th percentile) that lie above the 95. percentile in our study (1.27 µg/L).

**Table 6:** Metal exposure of recycling workers (mg/m^3^) in five german e-waste recycling sheltered workshops with reference to occupational exposure limits (OEL) in the breathing air and in biological material

Metal	Recycling workers Air monitoring, personal and stationary sampling			Recycling workers Biomonitoring		
	Highest concentration [mg/m^3^]	Limit value [mg/m^3^]	Limit value (type)	95.th- percentile	Limit value [µg/L] *[µg/g creatinine]	Limit value (type)
Aluminium	0.085 (Inh.)	10 (Inh.)	AGW (general dust limit)	9.77	50*	BGW
	0.0021 (Resp.)	1.25 (Resp.)	AGW (general dust limit)			
Antimony	0.00034 (Inh.)	0.5 (Inh.)	OEL (Austria)	2.03	0.2	BAR
	0.00088 (Resp.)					
Arsenic	0.000069 (Inh.)	0.00083 (Inh.)	AC	9.65	50	BLW
	0.000029 (Resp.)					
Beryllium	0.0000017	0.00014 (Inh.)				
0.00006 (Resp.)	AGW	0.05	0.05	BAR		
Cadmium	0.00023 (Inh.)			1.27	0.8	BAR
	0.0000094 (Resp.)	0.16 (Resp.)	AC			
Chromium	0.0014 (Inh.)	2 (Inh.)	AGW	0.25	0.6*	BAR
	0.00009 (Resp.)					
Cobalt	0.00031 (Inh.)			0.5	35	BLW
	0.000024 (Resp.)	0.005 (Resp.)	AC			
Nickel	0.0019 (Inh.)		AGW	2.96	3	BAR
	0.000088 (Resp.)	0.03 (Resp.)				
Mercury	0.0033	0.02	AGW	2.01	25*	BGW

The results of the air measurements (maximum concentrations) are placed in the context of German OELs for the inhalable (Inh.) or respirable (Resp.) dust fraction as time-weighted average (TWA) 8 h/shift (German: *Arbeitsplatzgrenzwerte*, AGW). Air measurements of carcinogenic metals are placed in the context of risk based german exposure levels (acceptable concentrations (AC)). The results of biomonitoring measurements (95.th percentile) are placed in the context of German biological limits and other reference values. These are biological limit values (*German: Biologischer Grenzwert*, BGW) the biological working substance reference values (*German: Biologischer Arbeitsstoffreferenzwert*, BAR) and biological guideline values (*German: Biologischer Leitwert*, BLW). See “Materials and methods” section for a detailed description of OELs and other reference values in this table

The individual background exposure of workers also contributes to urinary biomarker excretion. Certain dietary habits result in an increased intake of metals and may lead to a measurable increase of metal concentrations in urine. For example, seafood consumption is considered a key source of oral Hg intake (Bjørklund et al. 2017). Most participants in both groups in the present study consumed fish prior to the urine analysis. Dental amalgam fillings may also contribute to Hg exposure (Syversen and Kaur 2012). Almost half of participants reported having one or several dental amalgam fillings. Smoking is another important factor in non-occupational metal exposure. Smokers have a higher daily Cd intake than non-smokers through tobacco smoke (European Commission 2008). In our study, urine samples of smokers contained higher Cd concentrations than samples of non-smokers (0.25 µg/l, compared to 0.09 µg/l, geometric means). Beyond this, there were no indications of increased background exposure in study participants arising from the recorded non-occupational factors of influence.

Compared to the toxic metal exposure during e-waste recycling in conventional recycling facilities, exposure among workers in the studied sheltered workshops was low. In a recent literature review, Okeme et al. summarised toxic metal exposure in formal and informal e-waste recycling activities in various regions of the world (Okeme and Arrandale 2019). High exposure levels were particularly found in informal e-waste recycling operations where modern health, safety and environmental standards were absent, such as in Agbogbloshie, Ghana. However, high background exposures were also documented in institutions using modern occupational safety precautions. For instance, the results of a study on occupational metal exposure in the recycling of electrical waste from Sweden (Julander et al. 2014) showed that recycling workers were exposed to much higher air concentrations than office workers in the institutions studied (factor 10–30). Under these exposure conditions, recycling workers exhibited significantly higher blood/urine concentrations of certain metals (including Cd, Cr and Hg). For Sb and Hg, there was also a linear correlation between biomonitoring data and airborne metal dust concentrations. Zimmermann et al. showed that fluorescent lamp recycling operations in five French companies led to average Hg concentrations of 15.4 μg/m^3^ in the workshop air (Zimmermann et al. 2014). This Hg concentration exceeded the results of our study by a factor of 30 in the geometric mean and of 5 at peak exposure. Hg exposure during recycling of discarded energy-saving bulbs was also studied at pollutant collection sites in Germany (Paul et al. 2016). The Hg exposure of workers in this study (median, 0.03 μg/m^3^) was lower the exposure of workers in our study (median, 0.44 μg/m^3^), while the mean Hg concentration in the urine samples of the workers (mean 0.38 μg/g creatinine) is consistent with our results (mean 0.38 μg/g creatinine). One possible explanation for this finding is that there is no linear correlation between the inhaled Hg and the internal exposure (urine sample) at low air concentration levels.

The present cross-sectional study provides information on the toxic metal exposure of workers in sheltered workshops during e-waste recycling. Since the study design was cross-sectional, it only reflects the exposure of workers at a given time and the statements relate solely to that moment. Further limitations are the approximate nature of the activity descriptions and the lack of information on the duration of specific activities. Sample numbers were comparably low, but limited by the size of the facilities and the increased need for support of handicapped workers during measurements. Concerning data quality, our measurements resulted in a high number of samples below the LOD/LOQ (see “Results” section). We decided to apply the LOD (LOQ)/2 approach during basic statistical analysis (Hornung et. al 1990). This approach leads to conservative results overestimating the actual exposure of the recycling workers and do not allow the application of more sophisticated statistical analysis. Despite these limitations in data quality, this study, with its inclusion of air monitoring, biomonitoring and worker surveys, provides valuable information on occupational exposure to toxic metals among workers in sheltered workshops. It provides a basis for decision-makers for taking suitable safety measures in comparable facilities. Moreover, two specific measures to reduce exposure have been derived from the data: CCFL-tubes containing Hg should be immediately removed after disassembly of LCD screens and closed dust removal booths for pre-cleaning of CRT-devices contribute to further reduce workers metal exposure during e-waste recycling.

## Conclusion

Using a combined air- and biomonitoring approach we covered the major routes of occupational exposure of workers to toxic metals in sheltered workshops during recycling of electronic waste. We conclude that workers in these enterprises were only exposed to low amounts of toxic metals below current German occupational exposure levels, since adequate work methods and safety measures were established. Some working techniques resulted in elevated air concentrations of metals and should be avoided, including to storage of (broken) Hg-containing CFFL-tubes in open boxes next to the workplaces and the use of conventional vacuum cleaners during pre-cleaning of CRT-devices. These findings may provide a basis for decision-makers in establishing suitable safety measures in comparable facilities.

## Supplementary Information

Below is the link to the electronic supplementary material.

Supplementary file1 (DOCX 18 KB)

## Data Availability

The datasets generated during and/or analysed during the current study are available from the corresponding author on reasonable request.
