# Supplementary material for: Metal exposure of workers during recycling of electronic waste: a cross-sectional study in sheltered workshops in Germany
Source: Int Arch Occup Environ Health. 2021 Jan 24;94(5):935–44. doi: 10.1007/s00420-021-01651-9 (PMC8238705; doi:10.1007/s00420-021-01651-9)
Supplement: Supplementary file 1 — Supplementary file1 (DOCX 18 KB) [file 420_2021_1651_MOESM1_ESM.docx]

# Supplementary Material

## Validation of air sample laboratory methods

The analysis of the sample carriers encompassed the analytes aluminium, antimony, arsenic, cadmium, chromium, cobalt and nickel, and was performed at the Institute for Occupational Safety and Health of the German Social Accident Insurance in accordance with the validated IFA method 7808 “Metals and their compounds (ICP mass spectrometry)” (IFA 2013).

Samples were prepared using open-vessel hot block digestion with an acid mixture comprising 2 parts by volume 65% nitric acid (nitric acid; low-metal, content certified by manufacturer specifically for batch, e.g. 65% nitric acid, Suprapur®, Merck KGaA) and 1 part by volume 25% hydrochloric acid (hydrochloric acid; low-metal, content certified by manufacturer specifically for batch; e.g. 30% hydrochloric acid, Suprapur^®^, Merck KGaA) (IFA 2018). The samples were mixed with a defined volume (10 ml) of the aforementioned solution in a graduated vessel and boiled for two hours under reflux at 135°C in a thermostat-controlled hot block. The samples were then mixed with a defined amount of ultra-pure water (10 ml) (ρ ≥ 18.2 MΩ × cm at 25°C) and heated to boiling point. After the samples had been allowed to cool to room temperature, the volume was read; the samples were diluted (minimum 1:10), mixed with internal standard tellurium and lutetium and introduced to the analysis device using an autosampler. In order to determine the blank value, two filters from the same batch were subjected to the entire processing procedure with each digestion series and analysed in the same way as the air samples.

The analytes were quantified by inductively coupled plasma mass spectrometry (ICP-MS) (NexIon 350D, PerkinElmer LAS (Germany) GmbH) using multi-element standards as calibration solutions (multi-element calibration standard 3, 10 µg/ml & multi-element calibration standard 4, 10 µg/ml, PerkinElmer LAS (Germany) GmbH) in a concentration range of 0.01 µg/l to 50 µg/l, with internal standard added (see Table 7). For the elements arsenic and cadmium, a tellurium standard with a concentration of 15 µg/l in the measurement solution was used as the internal standard (tellurium plasma standard solution, 1000 µg/ml, Alfa Aesar). For the elements aluminium, antimony, beryllium, chromium, cobalt and nickel, a lutetium standard with a concentration of 2 µg/l in the measurement solution was used (lutetium ICP standard, 1000 mg/l, Merck KGaA). The analysis method was validated in accordance with DIN EN 32645 (2008), DIN EN 13890 (2010) and ISO 21832 (2018). The determination limits were set on each working day in accordance with the blank value method of DIN EN 32645 (2008) and varied between 0.017 and 2 µg/m³ depending on the analyte and the sampling air volume. To verify the correctness and stability of the analysis method used, periodical quality control samples were analysed. Therefore a multi-element standard (multi-element quality control standard, 100 mg/l for 33 elements for ICP, VWR) with a concentration of 0.1 µg/l, 1 µg/l and 10 µg/l of all analytes in the measurement solution was used. The quality control samples were added with the internal standards in the same way as the air samples and were matrix-adjusted. The measurement method used complies with the requirements regarding the performance of methods used to measure chemical working substances as stipulated in DIN EN 482 (2015), and complies with TRGS 402 (Committee on Hazardous Substances 2014).

**Table 7:** Validation parameters of laboratory methods for determining metal concentrations in air samples.

| Analyte | calibration range [µg/l] | Internal standard concentration of tellurium and lutetium in the measuring solution [µg/l] | limit of quantification (LOQ)* |
| --- | --- | --- | --- |
| Aluminium | 10-150 | 2 | 0.2 |
| Antimony | 0.01-5 | 2 | 0.17 |
| Arsenic | 0.01-5 | 15 | 0.026 |
| Beryllium | 0.01-1 | 2 | 0.17 |
| Cadmium | 0.1-10 | 15 | 0.017 |
| Chromium | 0.5-50 | 2 | 0.86 |
| Cobalt | 0.01-5 | 2 | 0.10 |
| Nickel | 0.1-10 | 2 | 0.39 |

*Relates to 1.2 m³ air sample volume and 20 ml digestion volume. The LOQ is recalculated each working day.

**References**

Committee on Hazardous Substances (2014) Technical Rule for Hazardous Substances (TRGS) 402: Identification and assessment of the risks from activities involving hazardous substances: inhalation exposure, German version: GMBl 2010 S. 231 253 v. 25.2.2010 [Nr. 12], last changed GMBl 10/2016.

Air monitoring methods in German language, 2019. In: The MAK‐Collection for Occupational Health and Safety 4 (2019) Nr. 4, S. 2391−2415. Wiley-VCH, Weinheim
https://onlinelibrary.wiley.com/doi/10.1002/3527600418.amsampaerod0020

DIN EN 32645 (2008) Chemical analysis - Decision limit, detection limit and determination limit under repeatability conditions - Terms, methods, evaluation. Beuth-Verlag, Berlin.

DIN EN 13890 (2010) Workplace exposure - Procedures for measuring metals and metalloids in airborne particles - Requirements and test methods; German version. Beuth-Verlag, Berlin

DIN EN 482 (2015): Workplace exposure - General requirements for the performance of procedures for the measurement of chemical agents; German version. Beuth-Verlag, Berlin

Institute for Occupational Safety and Health of the German Social Accident Insurance (IFA) (2013). Measurement of hazardous substances – IFA Folder. Determining of exposure to chemical and biological hazards 7808. Erich Schmidt Verlag, Berlin, loose-leaf

Institute for Occupational Safety and Health of the German Social Accident Insurance (IFA) (2018). Determining of exposure to chemical and biological hazards 6015. Erich Schmidt Verlag, Berlin, loose-leaf

ISO 21832 (2018) Workplace air – Procedures for determination of metals and metalloids in airborne particles. Ausgabe November 2018, Beuth-Verlag GmbH, Berlin
